# A Novel Noninvasive Approach Based on SPECT and EEG for the Location of the Epileptogenic Zone in Pharmacoresistant Non-Lesional Epilepsy

**DOI:** 10.3390/medicina55080478

**Published:** 2019-08-14

**Authors:** Karla Batista García-Ramó, Carlos A. Sanchez Catasus, Lilia Morales Chacón, Angel Aguila Ruiz, Abel Sánchez Corneaux, Pedro Rojas López, Jorge Bosh Bayard

**Affiliations:** 1 Nuclear Medicine Department, International Center for Neurological Restoration, 11300 Havana, Cuba; 2 Department of Nuclear Medicine and Molecular Imaging, University Medical Center Groningen, 9713 GZ Groningen, The Netherlands; 3 Department of Radiology, Division of Nuclear Medicine, University of Michigan, Ann Arbor, MI 48103, USA; 4 Neurophysiology Department, International Center for Neurological Restoration, 11300 Havana, Cuba; 5 Neuroinformatics Department, Cuban Neuroscience Center, 11300 Havana, Cuba

**Keywords:** EEG inverse solution, SPECT, SISCOM, epilepsy surgery, multimodal imaging

## Abstract

*Background and objectives*: The aim of this study is to propose a methodology that combines non-invasive functional modalities electroencephalography (EEG) and single photon emission computed tomography (SPECT) to estimate the location of the epileptogenic zone (EZ) for the presurgical evaluation of patients with drug-resistant non-lesional epilepsy. *Materials and Methods*: This methodology consists of: (i) Estimation of ictal EEG source imaging (ESI); (ii) application of the subtraction of ictal and interictal SPECT co-registered with MRI (SISCOM) methodology; and (iii) estimation of ESI but using the output of the SISCOM as a priori information for the estimation of the sources. The methodology was implemented in a case series as an example of the application of this novel approach for the presurgical evaluation. A gold standard and a coincidence analysis based on measures of sensitivity and specificity were used as a preliminary assessment of the proposed methodology to localize EZ. *Results*: In patients with good postoperative evolution, the estimated EZ presented a spatial coincidence with the resection site represented by high values of sensitivity and specificity. For the patient with poor postoperative evolution, the methodology showed a partial incoherence between the estimated EZ and the resection site. In cases of multifocal epilepsy, the method proposed spatially extensive epileptogenic zones. *Conclusions*: The results of the case series provide preliminary evidence of the methodology’s potential to epileptogenic zone localization in non-lesion drug-resistant epilepsy. The novelty of the article consists in estimating the sources of ictal EEG using SISCOM result as a prior for the inverse solution. Future studies are necessary in order to validate the described methodology. The results constitute a starting point for further studies in order to support the clinical reliability of the proposed methodology and advocate for their implementation in the presurgical evaluation of patients with intractable non-lesional epilepsy.

## 1. Introduction

About 20%–30% of people with epilepsy have resistance to treatment with antiepileptic drugs [1]. The surgical procedure is a therapeutic option for some of these patients with refractory drug-resistant epilepsy. The success of surgical treatment depends on the accurate and confident identification of the epileptogenic zone (EZ), its extension and its relationship with adjacent functional areas. This becomes a great challenge for patients with no or subtle magnetic resonance imaging lesions or with ambiguous electro-clinical signs. Various non-invasive methods to determine the surgery site, including clinical semiology, interictal/ictal video electroencephalography (EEG), magnetic resonance imaging (MRI) are essential. Single photon emission computed tomography (SPECT) and positron emission tomography (PET) are performed as additional tests in cases with “non-lesional” MRI and non-concordant electro-clinical findings. Also, intracranial EEG is often used to localize the area responsible for seizure, but this technique is invasive and cannot sample the activity from the whole brain. A deeper presurgical evaluation with non-invasive approaches and technologies is required in the attempt of identifying the EZ, the eloquent cortical areas and, consequently, the optimal resective surgical strategy.

The brain perfusion ictal and interictal single-photon emission computed tomography (SPECT) together with the subtraction of ictal and interictal SPECT co-registered with MRI (SISCOM) are valuable noninvasive tools in the presurgical evaluation of patients with medically intractable epilepsies. These images offer a high criterion of veracity in ictal onset detection represented by an increase in cerebral blood perfusion [2]. Previous studies have reported a sensitivity of interictal SPECT around 44%, and ictal over 97% in temporal lobe epilepsy, contrary to a sensitivity of 66% in ictal and 40% interictal in extra-temporal epilepsy [3]. On the other hand, ictal electroencephalography (EEG) source imaging (ESI) allow us to infer the configuration of neuronal sources responsible for ictal activity. Several methods and inverse solutions have been proposed for source imaging of epileptiform EEG activity [4,5,6]. Due to the underdetermined nature of the inverse problem, each method operates with specific additional constraints in order to localize the source. The solution can only be found using additional information (spatial or temporal) on the sources of current either as a function of the geometric or physiological properties of the brain, which can be included as restrictions [7]. For a review on these methods see [8].

The objective of this article is to propose a methodology that combines non-invasive functional modalities EEG and SPECT in identifying and localizing the epileptogenic zone for the presurgical evaluation of patients with drug-resistant non-lesional epilepsy. This article presents a case series as an example of the application of this novel approach. A gold-standard is established as an initial assessment of methodology’s potential to localize EZ based on Engel scale and spatial coincidence of estimated EZ with the resection site. No previous studies were found using SISCOM output as a spatial restriction for the estimation of EEG sources.

This article illustrates the methodology follow up for the presurgical evaluation in the first Cuban comprehensive surgery program carried out at the International Center for Neurological Restoration in Havana, Cuba [9].

## 2. Materials and Methods

### 2.1. Case Series

The proposed methodology is applied, retrospectively, to five patients (Table 1) diagnosed with drug resistant refractory epilepsy with complex partial seizures refractory to pharmacological treatment. These patients belong to the aforementioned epilepsy research protocol. For the presurgical evaluation, an ictal and inter-ictal video electroencephalogram was performed, as well as magnetic resonance studies, neuropsychological evaluation and semiology of the seizures. Taking into account that MRI showed no visible epileptogenic lesion and discordant results of electro-clinical evaluation without a clear EZ hypothesis, an ictal study and an inter-ictal study of brain perfusion SPECT was performed.

Postsurgical outcome one year after surgery was evaluated according to the modified Engel classification, Engel I outcome patients (favorable outcome) versus Engel II–IV outcome patients (unfavorable outcome). Additionally, each patient who underwent surgery had an MRI one year after surgery.

Patients were included in this study if they (1) had undergone both ictal and interictal SPECT imaging before surgery, (2) had a volumetric preoperative MRI, and (3) had a volumetric postsurgical MRI 1 year after surgery.

All the procedures followed the rules of the Declaration of Helsinki of 1975 for human research, and the study was approved by the scientific and ethics committee from the International Center for Neurological Restoration (CIREN37/2012).

### 2.2. SPECT and SISCOM

A brain perfusion SPECT was performed for all subjects, with the same gamma camera, and under the same conditions. Each patient underwent two studies (Ictal and Inter-ictal) of brain perfusion SPECT using 99mTc-ethylene-cysteine dimer (ECD). In both studies during the administration of the radiopharmaceutical, the patient remained monitored by EEG. For inter-ictal SPECT, the dose is administered with the patient at rest and with a seizure-free period of more than 24 h. For ictal SPECT, the radiopharmaceutical was injected when the onset of a crisis with EEG is recognized. SPECT image acquisition was performed using a double-headed gamma camera (SMV DST-XLi, Buc Cedex, France) equipped with a fan-beam collimator. One hundred and twenty-eight projections in a circular orbit of 360° per subject were collected in matrices of 128 × 128 pixels and with an energy window of 20% centered on the photopeak of 99 mTc. Tomographic 3D reconstruction was performed using a DROSEM algorithm. The SISCOM methodology was carried out, according to O’Brien et al. [10]. Ictal and interictal SPECT studies were co-registered using an automatic registration algorithm based on mutual information using the registration module of Statistical Parametric Mapping (SPM version 8; Wellcome Trust Centre for Neuroimaging, London, UK), implemented in Matlab (R2014a; The MathWorks Inc., Natick, MA, USA). Each SPECT volume was multiplied by a binary mask to remove the extracerebral activity. The ictal and spatially co-registered interictal images were then normalized for global brain counts within each scan. The transformed, normalized interictal images were subtracted from the normalized ictal image to create an image where the value for each pixel represents the intensity difference between the two data sets. The difference image was smoothed using a 3D-Gaussian smoothing kernel (full width at half maximum = 12 mm) and transformed into a z-score using the mean and standard deviation (SD) of the differences in all brain voxels. (z-score = 2). For co-registration with the MRI scan, the cerebral surface of the MRI volume was segmented from the extracerebral structures. The cerebral surface of the binary ictal SPECT was then matched to the cerebral surface of the binary MRI. The resulting transformation matrix was applied to the subtraction SPECT to co-register it to the cerebral surface of the MRI.

### 2.3. Inverse Solution from Ictal EEG

The cortical generators of EEG measurements can be estimated by solving an inverse imaging problem where the unknown sources are distributed on an individual’s cortex. The methodology followed for the estimation of the inverse solution of ictal EEG using EEGLAB MATLAB toolbox consists of:

(1) source space definition in order to model the link between the EEG measurements and cortical activity. Because the generators of the signals are believed to be mainly cortical, the source space is given- by the triangularized inner cortical surface (gray matter/white matter interface), segmented from individual patient’s T1-weighted 3D MRI;
(2) the spatial co-registration of the measurement electrodes to the using the Montreal Neurological Institute (MNI; Montreal, Quebec, Canada) MNI152 template using translation and rotation;
(3) forward modeling of the cortical currents: To model the relationship between the source space and the EEG measurements at the sensor level. The source space, the 3D electrode locations, and the individually defined boundaries are then combined to characterize the electric field propagation with a three-compartment boundary element method (BEM) [11]; and finally
(4) inversion of the model for source estimation using the Multiple Sparse Priors (MSP) algorithm [4].

### 2.4. SISCOM as Spatial Priors for Inverse Solution of Ictal EEG

Currently, the available priors in ESI gain from MRI, functional MRI and PET [12,13]. In this paper we propose to use SPECT as a way to constrain the EEG source location on the assumption that a subset of the neuronal activity is detectable by both modalities. Thus, the SISCOM result can be used to inform the source localization method about the location of the sources. SISCOM images reveal ictal hyperperfusion, which should correspond to the onset zone of epileptogenic crisis. This restriction is based on the conjecture that synaptic currents that generate EEG signals also impose metabolic demand, which leads to an increase in cerebral blood flow as measured by SPECT. Steps 1 to 3 are the same as above but, in addition to the mathematical priors, the ictal hyperperfusion derived from SISCOM is considered to constraint ictal EEG source location:

(1) source space definition in order to model the link between the EEG measurements and cortical activity. Because the generators of the signals are believed to be mainly cortical, the source space is given- by the triangularized inner cortical surface (gray matter/white matter interface), segmented from individual patient’s T1-weighted 3D MRI;
(2) the spatial co-registration of the measurement electrodes to the using the Montreal Neurological Institute (MNI; Montreal, Quebec, Canada) MNI152 template using translation and rotation;
(3) forward modeling of the cortical currents: To model the relationship between the source space and the EEG measurements at the sensor level. The source space, the 3D electrode locations, and the individually defined boundaries are then combined to characterize the electric field propagation with a three-compartment boundary element method (BEM) [11];
(4) Functional area definition using SPECT: To localize the functional areas using SISCOM methodology that will be used to constrain the inverse procedure;
(5) SISCOM-informed inverse modeling of the cortical currents: To use the ictal hyperperfusion area derived from SISCOM to constrain the estimation of the cortical activity generating the EEG measurements using MSP algorithm.

### 2.5. Coincidence Analysis

For the present case series, a coincidence analysis was performed as an initial evaluation of methodology. The three EZ proposed by the methodology: SISCOM, ictal ESI and ictal ESI using SISCOM as a prior, were co-registered with the post-surgery anatomical image T1. Also, the results were co-registered with AAL atlas [14] to identify anatomical regions corresponding to surgical resection. A gold-standard was established to evaluate the veracity of the estimated EZ. Engel I outcome (crisis-free) one year after surgery and a spatial intersection of the hypothetical EZ with the resection site was established as a gold-standard. The spatial concordance was evaluated using sensitivity and specificity measures defined as:

(1) $$\mathrm{Sensitivity}=\frac{\mathrm{Truepositives}}{\mathrm{Truepositives}+\mathrm{Falsenegatives}}$$

(2) $$\mathrm{Specificity}=\frac{\mathrm{Truenegatives}}{\mathrm{Truenegatives}+\mathrm{Falsepositives}}$$

A low sensitivity would indicate many false negatives. On the other hand, the specificity reflects the methodology’s capacity to not reporting false positives.

## 3. Results

**Figure 1:**
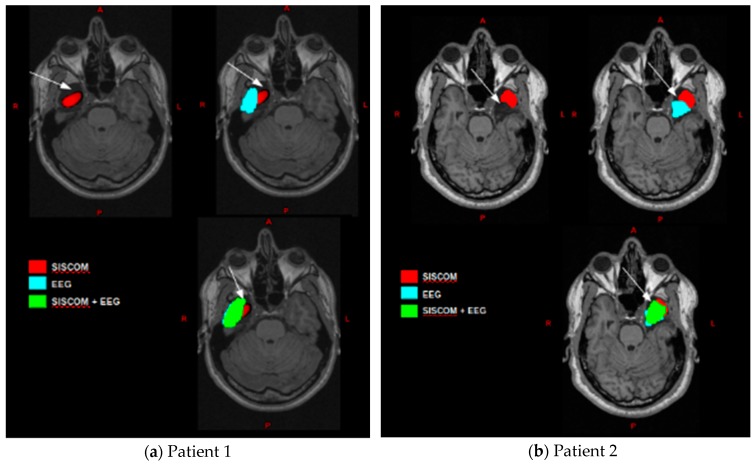
Co-registration of post-operative T1 with EZ images obtained by: subtraction of ictal and interictal SPECT co-registered with MRI (SISCOM) (in red), ictal EEG source imaging (ESI) (blue) and ictal ESI using SISCOM as a prior (green); (**a**) for patient 1; and (**b**) for patient 2. A white arrow indicates resection zone.

Five patients were evaluated with this methodology, and three patients were subjected to surgical treatment. Two patients (patients 1 and 2) had a favorable outcome (Engel I). In these two cases, the EZ estimated by the proposed methodology shows spatial coincidence with the resection site represented by high values of sensitivity, as well as specificity (Table 2). For both patients, SISCOM result consisted of a single confined region that correspond to the resection site but without covering it completely (Figure 1 in red). Ictal ESI (Figure 1 in blue) consisted in a region more extended than SISCOM outcome, but also includes voxels that corresponded to false positives. Ictal ESI using SISCOM as a prior revealed a region, including more voxels of the resection site than SISCOM and ictal ESI individually, but also false positives, due to ictal ESI outcome (Figure 1 in green). These results are quantified in the coincidence analysis, and high sensitivity values were obtained for ictal ESI (82.1%), SISCOM (65%) and ictal ESI + SISCOM (92.6%), and only high specificity for the SISCOM case (99.9%) (Table 2).

**Figure 2:**
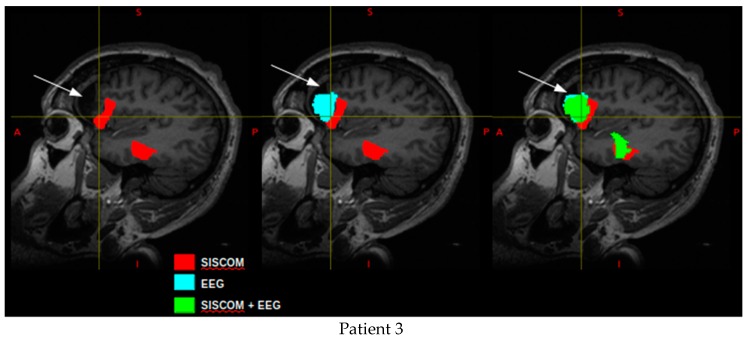
EZ estimated by the proposed methodology and co-registered to post-operative T1-weighted MRI: SISCOM (in red), ictal ESI (blue) and ictal ESI using SISCOM as a prior (green) for patient 3. The white arrow indicates the resection site.

Patient 3 underwent surgery for the resection of the right frontal lobe. Unfortunately, the patient had an unfavorable outcome (Engel III), which indicates recurrence seizures. Two regions were identified with SISCOM methodology (Figure 2 in red), one that most of the voxels remained posterior to resection site; and the other corresponding to para-hippocampus and fusiform gyrus. The ictal ESI revealed voxels that coincide to the resection site but also voxels that were posterior to it (Figure 2 in blue) and voxels of the frontal pole in the opposite hemisphere. The ictal ESI using SISCOM as a prior proposed voxel that coincide with the resection site but also voxels that remain posterior to it (Figure 2 in green); in addition to the voxels that correspond to the para-hippocampus and fusiform gyrus, due to SISCOM restriction. Low values of both sensitivity and specificity were obtained for this patient (Table 2).

**Figure 3:**
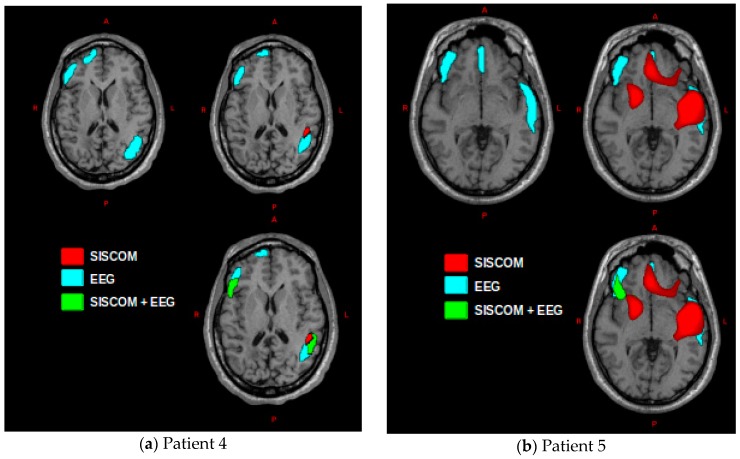
EZ estimated by the proposed methodology and co-registered to T1-weighted MRI: SISCOM (in red), ictal ESI (blue) and ictal ESI using SISCOM as prior (green); (**a**) for patient 4; and (**b**) for patient 5.

Patients 4 and 5 were diagnosed with extra-temporal epilepsy and, as the rest of patients, did not present appreciable lesions in the structural MRI. Both cases were diagnosed with multifocal epilepsy, and they were not subjected to surgical intervention. The estimation of the sources from ictal ESI revealed extensive cortical zones that includes bilateral temporal and frontal lobes for both cases (Figure 3 in blue). SISCOM results showed confined regions in the right frontal and left temporal for patient 4 (Figure 3a in red) and in frontal and temporal lobes bilaterally for patient 5 (Figure 3b in red). Ictal ESI incorporating SISCOM as a prior (Figure 3 in green) consisted of scattered zones in both hemispheres and lobes (temporal and frontal). Unlike the previous cases, it is not possible to determine spatially confined EZ. The methodology proposes scattered and diverse EZ, which is consistent with the diagnosis of multifocal epilepsy.

## 4. Discussion

In the present work, we propose a methodology that combines different functional neuroimaging techniques to identify epileptogenic zone (EZ) in patients with drug-resistant non-lesional epilepsy. Single-photon emission computed tomography (SPECT), subtraction of ictal and interictal SPECT co-registered with MRI (SISCOM), and ictal electroencephalography (EEG) source imaging (ESI) are complementary diagnostic methods in the localization of seizure zone when MRI is negative. The novelty of the article consists in estimating the sources of ictal EEG using SISCOM result as a prior for the inverse solution. There are no research records that use SPECT-SISCOM as a prior for ictal ESI in the consulted literature. A case series is presented to implement the described methodology. A gold-standard of value I on the Engel scale and spatial coincidence of estimated EZ with the resection site was established for postsurgical evaluation of patients. The spatial coincidence analysis allows a preliminary quantitative evaluation of the methodology based on sensitivity and specificity measurements.

Five patients were evaluated with this methodology, and three patients were subjected to surgical treatment. Two patients had favorable outcome (Engel I). In these two cases (patients 1 and 2) the methodology proposed EZ that coincide spatially with the resection site represented by high values of sensitivity, as well as specificity (Table 2). The low specificity values of the ictal ESI are due to false positives.

The third patient underwent surgery did not have a favorable outcome with continued seizures ensue (Engel III). The methodology reported EZ that were not included in the resection site for this patient, instead were localized posterior it. For this particular case, low values of both sensitivity and specificity were obtained.

The remaining two patients did not undergo surgery because they were diagnosed with multifocal epilepsy after the presurgical evaluation. The implementation of our methodology for these two patients proposed diverse and dispersed EZ that cover both hemispheres. This result demonstrates that the methodology can be used not only in the cases of focal non-lesional epilepsy for determination of EZ, but also in multifocal cases.

The proposed methodology estimates EZ by ictal ESI, SISCOM and ictal ESI using SISCOM as prior. For the present case series, which was an initial test of the methodology, shows that the highest values of the sensitivity of localizing EZ were obtained for ictal ESI using SISCOM as a prior. Due to the spatial location of the neuronal sources of the scalp recorded EEG activity cannot be conclusively determined (inverse problem has no unique solution) by introducing reasonable priors from other modalities, inverse solution methods reveal the most probable sources and communication structures. Further studies are necessary in order to conclude that combining EEG and SPECT allows less false negatives, which would contribute to a better surgical outcome. Otherwise, SISCOM has the highest specificity providing reliability by false positives. The objective of this work is not to evaluate the veracity of the individual techniques used (ESI or SISCOM) to identify EZ in drug-resistant epilepsy, which has been the goal of some publications [15,16,17,18]. The disadvantages of each individual technique are balanced by establishing a methodology that uses and combines different functional modalities. ESI methods provide an estimate of the resulting current density at each voxel that is a combination of the real source values over all the voxels of the brain, which causes a remarkable distortion in the estimation of sources. Therefore, ESI methods have the disadvantage of proposing spurious sources, as well as a high level of spatial dispersion; and it is also ineffective in detecting the activation of deep structures. This explains the presence of false positives and the inability of this method to detect deep structures, such as amygdala and hippocampus. These drawbacks are not present in the case of SISCOM methodology. However, the accuracy of the SISCOM has various seizure-related factors that affect the localization of EZ; and the most significant one is timing tracer injection [19]. The delayed injection may detect secondary hyper-perfusion, due to seizure spread, non-localized findings or even false locations of EZ. The rapid propagation of ictal activity in certain seizures makes it difficult to select the signals corresponding to the onset. SISCOM as a prior for ESI includes information about structures not detectable with scalp EEG, because they are too deep. However, the more reliant SISCOM, the more trust-able, would be the source estimations.

The major limitation of this study is the small sample size. Therefore, we anticipate that future studies with increased sample size will be able to validate the proposed methodology. Nonetheless, the main value of this study is to outline a novel noninvasive approach combining MRI, SPECT and EEG for the location of the epileptogenic zone in pharmacoresistant non-lesional epilepsy.

From the above discussion, it can be concluded that the proposed methodology may contribute to epileptogenic zone localization in non-lesion drug-resistant epilepsy. The case series, although limited by small sample size, suggests that combining ictal ESI and SISCOM has higher predictive value than individual techniques for good outcomes. Before implementing SISCOM methodology or ictal ESI methods, care must be taken their advantages and disadvantages. However, the fusion of the different methodologies is helpful and give better results than implement only individual techniques.

## 5. Conclusions

The main aim of this paper is to a new methodology based on the integration of non-invasive functional neuroimages to an accurate estimation of the source generating epileptiform discharges for the presurgical evaluation of patients with drug-resistant focal epilepsy. The methods have been oriented to the identification of the epileptogenic zone by the processing of ictal EEG and ictal and inter-ictal SPECT. The methodology proposed offers relevant information about EZ in both temporal and extra-temporal, focal and multifocal epilepsy. The major limitation of this study is the small sample size. The case series results provide future study directions in order to validate the proposed methodology and advocate for their implementation in the presurgical evaluation of patients with intractable non-lesional epilepsy. The fusion of different non-invasive functional techniques on the magnetic resonance imaging of the patient provides valuable information in identifying and localizing the epileptogenic zone to be considered as a surgical resection site. Localizing information of one modality may enhance detection of subtle abnormality in another modality.

## Figures and Tables

**Table 1:** Demographic and clinical data of the sample.

Patient	Age	Gender	Epilepsy Type	EZ Hypothesis	Surgical Site	Post-Operative Outcome
						Engel Class
1	35	Male	Temporal	Right temporal	Right temporal	I
2	38	Male	Temporal	Left temporal	Left temporal	I
3	17	Male	Extra-temporal	Right frontal	Right frontal	III
4	39	Female	Extra-temporal	Right frontal and left temporal	No surgery	- ^1^
5	29	Male	Extra-temporal	Bilateral fronto-temporal	No surgery	- ^1^

^1^ Patients not operated. EZ, epileptogenic zone.

**Table 2:** Coincidence analysis of estimated EZ with the resection site.

Patient	Sensibility			Specificity		
	SISCOM	Ictal ESI	Ictal ESI + SISCOM	SISCOM	Ictal ESI	Ictal ESI + SISCOM
1	65%	82.1%	92.6%	99.9%	46.4%	61.7%
2	55.2%	58.7%	83.4%	99.9%	43.9%	65%
3	3.5%	44.6%	49%	52.9%	47.4%	48.5%

## References

[1] Weaver D.F., Pohlmann-Eden B. (2013). Pharmacoresistant epilepsy: Unmet needs in solving the puzzle(s). Epilepsia.

[2] Chen T., Guo L. (2016). The role of SISCOM in preoperative evaluation for patients with epilepsy surgery: A meta-analysis. Seizure.

[3] Anyanwu C., Motamedi G.K. (2018). Diagnosis and Surgical Treatment of Drug-Resistant Epilepsy. Brain Sci..

[4] Friston K., Harrison L., Daunizeau J., Kiebel S., Phillips C., Trujillo-Barreto N., Henson R., Flandin G., Mattout J. (2008). Multiple sparse priors for the M/EEG inverse problem. NeuroImage.

[5] Nara S., Sheoran P. (2018). Advancements in EEG source localisation methods. Int. J. Med. Eng. Inform..

[6] Biscay R.J., Bosch-Bayard J.F., Pascual-Marqui R.D. (2018). Unmixing EEG Inverse Solutions Based on Brain Segmentation. Front. Neurosci..

[7] Lei X., Wu T., Valdes-Sosa P.A. (2015). Incorporating priors for EEG source imaging and connectivity analysis. Front. Neurosci..

[8] Grech R., Cassar T., Muscat J., Camilleri K.P., Fabri S.G., Zervakis M., Xanthopoulos P., Sakkalis V., Vanrumste B. (2008). Review on solving the inverse problem in EEG source analysis. J. Neuroeng. Rehabil..

[9] Morales Chacon L.M., Garcia Maeso I., Baez Martin M.M., Bender Del Busto J.E., Garcia Navarro M.E., Quintanal Cordero N., Estupinan Diaz B., Lorigados Pedre L., Valdes Yerena R., Gonzalez J. (2018). Long-Term Electroclinical and Employment Follow up in Temporal Lobe Epilepsy Surgery. A Cuban Comprehensive Epilepsy Surgery Program. Behav. Sci. Basel Switz..

[10] O’Brien T.J., O’Connor M.K., Mullan B.P., Brinkmann B.H., Hanson D., Jack C.R., So E.L. (1998). Subtraction ictal SPET co-registered to MRI in partial epilepsy: Description and technical validation of the method with phantom and patient studies. Nucl. Med. Commun..

[11] Hamalainen M.S., Sarvas J. (1989). Realistic conductivity geometry model of the human head for interpretation of neuromagnetic data. IEEE Trans. Biomed. Eng..

[12] Daunizeau J., Grova C., Mattout J., Marrelec G., Clonda D., Goulard B., Pelegrini-Issac M., Lina J., Benali H. (2005). Assessing the relevance of fMRI-based prior in the EEG inverse problem: A bayesian model comparison approach. IEEE Trans. Signal Process..

[13] Luessi M., Babacan S.D., Molina R., Booth J.R., Katsaggelos A.K. (2011). Bayesian symmetrical EEG/fMRI fusion with spatially adaptive priors. NeuroImage.

[14] Tzourio-Mazoyer N., Landeau B., Papathanassiou D., Crivello F., Etard O., Delcroix N., Mazoyer B., Joliot M. (2002). Automated anatomical labeling of activations in SPM using a macroscopic anatomical parcellation of the MNI MRI single-subject brain. Neuroimage.

[15] Chacón L.M.M., Sánchez-Catasús C.A., Quincoses O.T., Pedre L.L., Dierckx R.A.J.O. (2014). Nuclear Medicine Neuroimaging and Electromagnetic Source Localization in Nonlesional Drug-Resistant Focal Epilepsy. PET and SPECT in Neurology.

[16] Beniczky S., Rosenzweig I., Scherg M., Jordanov T., Lanfer B., Lantz G., Larsson P.G. (2016). Ictal EEG source imaging in presurgical evaluation: High agreement between analysis methods. Seizure.

[17] Khosropanah P., Ramli A.R., Lim K.S., Marhaban M.H., Ahmedov A. (2018). Fused multivariate empirical mode decomposition (MEMD) and inverse solution method for EEG source localization. Biomed. Eng. Biomed. Tech..

[18] Long Z., Hanson D.P., Mullan B.P., Hunt C.H., Holmes D.R., Brinkmann B.H., O’Connor M.K. (2018). Analysis of Brain SPECT Images Coregistered with MRI in Patients with Epilepsy: Comparison of Three Methods. J. Neuroimaging Off. J. Am. Soc. Neuroimaging.

[19] Lee J.Y., Joo E.Y., Park H.S., Song P., Young Byun S., Seo D.W., Hong S.B. (2011). Repeated ictal SPECT in partial epilepsy patients: SISCOM analysis. Epilepsia.

